# Design and Optimization of Linking Matrix for a JSCC System Based on DP-LDPC Codes

**DOI:** 10.3390/e25081145

**Published:** 2023-07-31

**Authors:** Yijie Lv, Shaohua Hong, Lin Wang

**Affiliations:** Department of Information and Communication Engineering, Xiamen University, Xiamen 361005, China; lvsejianke@126.com (Y.L.); wanglin@xmu.edu.cn (L.W.)

**Keywords:** joint source-channel coding, joint protograph extrinsic information transfer, decoding threshold, structural constraint

## Abstract

Although extensive optimization of encoding and decoding schemes for joint source-channel coding (JSCC) systems has been conducted, efficient optimization schemes are still required for designing and optimizing the linking matrix between variable nodes of the source code and check nodes of the channel code. A scheme has been proposed for design and optimization of linking matrix with multi-edges by analyzing the performance of the JSCC system using the joint protograph extrinsic information transfer algorithm to calculate decoding thresholds. The proposed scheme incorporates structural constraints and is effective in designing and optimizing the multi-edges in linking matrix for the JSCC system. Experimental results have demonstrated that the designed and optimized linking matrix significantly improves the performance of the JSCC system. Furthermore, the proposed scheme reduces the complexity of the solution space for the optimized example.

## 1. Introduction

Shannon introduced a channel code that ensures reliable communication, provided that the information rate of the code is below the channel capacity [1]. However, the application of Shannon theory in separate source-channel optimization imposes limitations on the performance of practical digital communication systems [2,3,4]. This results in elevated decoding errors due to the underutilization of residual redundancy present at the output of the source decoder [5]. Moreover, the source-channel separation scheme, which involves sequential source decoding and channel decoding operations without the exchange of extrinsic information, leads to a degradation in performance [6].

The joint source-channel coding (JSCC) system solves the drawbacks of the standard source-channel separation coding system described above [5,6]. In order to improve the performance of the JSCC system, a technique called joint protograph extrinsic information transfer (JPEXIT) chart has been used in conjunction with two tandem protograph low-density parity-check (LDPC) codes [7,8]. Two tandem protograph LDPC codes in combination can facilitate hardware implementation in a structure that is also referred to as double protograph LDPC (DP-LDPC) codes [9,10]. Another JSCC method based on concatenated spatially coupled low-density parity-check codes has also been proposed [11]. A study has investigated the relationship between the check nodes (CNs) of the source and the variable nodes (VNs) of the channel [12]. The performance of the JSCC system has been enhanced through the utilization of a multi-objective optimization technique that considers the optimization of single-edge in linking matrix [13]. Through an analysis of the linking matrix between VNs in the source code and CNs in the channel code (i.e., $B_{L2}$), it has been confirmed that multi-edges in linking matrix provide a significant improvement to the performance of the JSCC system compared to single-edge in linking matrix [14,15,16,17]. Although it has been demonstrated that the presence of linking matrix with multi-edges can effectively improve the performance of JSCC systems, existing design and optimization schemes have only utilized partially multi-edges in linking matrix and lack an effective optimization scheme.

The main contributions of this paper are as follows. The effect of the linking matrix on the performance of JSCC systems is analyzed by calculating the decoding threshold through the joint protograph extrinsic information transfer algorithm. A scheme with structural constraints has been proposed for designing and optimizing linking matrix with multi-edges. The proposed optimization scheme for the linking matrix effectively improves the performance of the JSCC system while reducing the complexity of the search space.

The organizational structure of this paper is as follows: Section 2 provides a detailed introduction to the JSCC system. Section 3 presents a scheme for the design and optimization of linking matrix. Section 4 presents the experimental results and the summary. Finally, the conclusions of the experiments in this paper are given.

## 2. JSCC System Description

**Figure 1:**
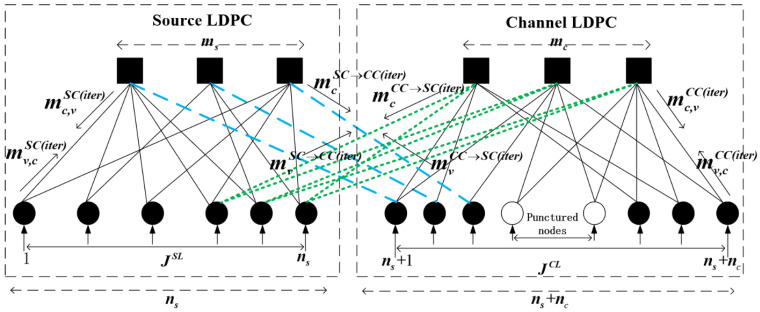
JSCC Tanner.

JSCC system employs a combination of source and channel codes to compress data and ensure reliable transmission. The system utilizes double LDPC codes, with DP-LDPC codes optimizing the performance of the waterfall and error floor [14]. The JSCC matrix representation is defined as (1).

(1) $$B_{J}=\left[\begin{array}{cc} B_{S} & B_{L1}\\ B_{L2} & B_{C} \end{array}\right],$$

where $B_{J}$ has a dimension of $\left(m_{s}+m_{c}\right)\cdot \left(n_{s}+n_{c}\right)$, the source code matrix $B_{S}$ has a dimension of $m_{s}\cdot n_{s}$, the channel code matrix $B_{C}$ has a dimension of $m_{c}\cdot n_{c}$, the $B_{L1}$ has a dimension of $m_{s}\cdot n_{c}$, and the $B_{L2}$ has a dimension of $m_{c}\cdot n_{s}$. The Tanner graph represented by the joint base matrix $B_{J}$ is depicted in Figure 1. The figure contains eight types of mutual information.

### 2.1. Encoder

The given source sequence is assumed to be independently and identically distributed according to a binomial Bernoulli distribution with probability *p*, where *p* (p≠1/2) represents the probability of a ’1’ occurring. The entropy of the source can be calculated using the formula:

(2) $$H=-p\log_{2}p-\left(1-p\right)\log_{2}\left(1-p\right),$$

Two tandem LDPC codes are used to represent the JSCC system.

(3) $$c=\left[pt,o\right]\cdot G_{LC}=\left[pt,pt\cdot H_{SC}^{T}\right]\cdot G_{LC},$$

where pt is the source output, *o* is the source compressed sequence, $G_{LC}(\left(n_{c}+l-m_{c}\right)\cdot$ $\left(n_{c}+l\right))$ (the quantities within the parentheses denote the dimensions or sizes of the matrices) is the generation matrix obtained from the algebraic transformation of the check matrix $H_{LC}\left(m_{c}\cdot \left(n_{c}+l\right)\right)$, and *l* is the number of columns in $B_{L2}$ that contain non-zero elements. The two tandem codes are under the following condition

(4) $$n_{c}-m_{c}=m_{s}.$$

### 2.2. Decoder

Simulation of binary sequence under additive white Gaussian noise (AWGN) channel after binary phase-shift keying (BPSK) modulation. The joint belief propagation (JBP) algorithm uses the sequence on the receiver side to recover the sequence on the sender side. The initial source information $J^{SL}$ is given by ln((1−p)/p). The initial channel information is $J^{CL}=2y/\sigma^{2}$, where $y=\left(1-2s_{i}\right)+n_{G}$, and $n_{G}∼\left(0,\sigma^{2}\right)$ is Gaussian noise.

## 3. Design and Optimization

### 3.1. JEXIT Analysis

To analyze the effect of $B_{L2}$ on the performance of JSCC system, the source code, the channel code, and $B_{L1}$ are fixed. The $e_{L2}^{i,j}$ term in (5) indicates the presence of a link between the *j*-th VN of the source code and the *i*-th CN of the channel code.

(5) $$B_{L2}=\left[\begin{array}{cccccccc} e_{L2}^{1,1} & e_{L2}^{1,2} & e_{L2}^{1,3} & e_{L2}^{1,4} & e_{L2}^{1,5} & e_{L2}^{1,6} & e_{L2}^{1,7} & e_{L2}^{1,8}\\ e_{L2}^{2,1} & e_{L2}^{2,2} & e_{L2}^{2,3} & e_{L2}^{2,4} & e_{L2}^{2,5} & e_{L2}^{2,6} & e_{L2}^{2,7} & e_{L2}^{2,8}\\ e_{L2}^{3,1} & e_{L2}^{3,2} & e_{L2}^{3,3} & e_{L2}^{3,4} & e_{L2}^{3,5} & e_{L2}^{3,6} & e_{L2}^{3,7} & e_{L2}^{3,8} \end{array}\right]$$

**Figure 2:**
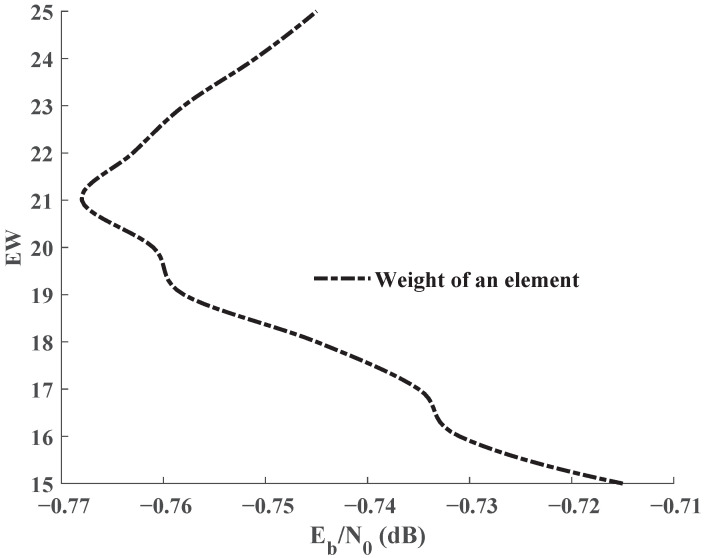
Decoding thresholds for an element with different weights in $B_{L2}$.

The joint base matrix in Equation (6) is composed of the source matrix $B_{S}$, the channel matrix $B_{C}$, $B_{L1}$ and $B_{L2}$. The decoding threshold is −0.568 dB when $B_{L2}$ is an all-zero matrix. As the weight of an element in $B_{L2}$ increases, the decoding threshold variation is depicted in Figure 2. The decoding threshold decreases as the weight of the element increases, and reaches a minimum when the weight increases to 21. After that, the decoding threshold increases as the weight of the element increases.

(6) $$B_{J}=\left[\begin{array}{ccccccccccccc} 3 & 1 & 3 & 1 & 3 & 1 & 1 & 1 & 0 & 0 & 0 & 1 & 0\\ 1 & 2 & 1 & 3 & 1 & 3 & 1 & 2 & 0 & 0 & 0 & 0 & 1\\ e_{L2}^{1,1} & e_{L2}^{1,2} & e_{L2}^{1,3} & e_{L2}^{1,4} & e_{L2}^{1,5} & e_{L2}^{1,6} & e_{L2}^{1,7} & e_{L2}^{1,8} & 1 & 0 & 1 & 2 & 0\\ e_{L2}^{2,1} & e_{L2}^{2,2} & e_{L2}^{2,3} & e_{L2}^{2,4} & e_{L2}^{2,5} & e_{L2}^{2,6} & e_{L2}^{2,7} & e_{L2}^{2,8} & 0 & 1 & 0 & 3 & 1\\ e_{L2}^{3,1} & e_{L2}^{3,2} & e_{L2}^{3,3} & e_{L2}^{3,4} & e_{L2}^{3,5} & e_{L2}^{3,6} & e_{L2}^{3,7} & e_{L2}^{3,8} & 1 & 1 & 2 & 0 & 2 \end{array}\right]$$

The decoding thresholds of different elements in $B_{L2}$ are listed in Table 1 when all elements have a weight of 5. From Table 1, it can be concluded that the addition of edge connections in $B_{L2}$ may not necessarily enhance the waterfall region of the JSCC system. In actuality, adding elements to the linking matrix may lead to the degradation of the system performance. The analysis also reveals that the third row of $B_{L2}$ is a critical area where adding edge connections may cause performance degradation. Therefore, to simplify the solution space and optimize the performance of JSCC system, it is advisable to avoid adding edge connections in the third row of $B_{L2}$.

### 3.2. Design and Optimization

The following structural constraints need to be satisfied for the design and optimization of the linking matrix:

(7) $$\begin{array}{c} Condition\;1:\frac{EW_{r}}{n_{s}}=E_{p}\leq 1, \end{array}$$

where the number of non-zero elements in the row is $EW_{r}$, the number of columns in the matrix is $n_{s}$, and the probability of non-zero elements in a specific row of the matrix is $E_{p}$.

(8) $$\begin{array}{c} Condition\;2:\frac{EW_{D}}{D}=ED_{p}<1, \end{array}$$

where the number of non-zero elements in the matrix is $EW_{D}$, the dimension of the matrix is *D*, and the probability of non-zero elements in the matrix is $ED_{p}$.

(9) $$\begin{array}{c} Condition\;3:\sum_{i=1}^{n_{s}}RW_{i}\leq RW_{\max}\leq \left(m_{s}+n_{s}+m_{c}+n_{c}\right), \end{array}$$

where the weight of the *i*-th element in the row is $RW_{i}$ and the maximum value of the row weight is $RW_{\max}$.

(10) $$\begin{array}{c} Condition\;4:\sum_{j=1}^{m_{c}}CW_{j}\leq CW_{\max}\leq n_{s}, \end{array}$$

where the weight of the *j*-th element in the column is $CW_{j}$ and the maximum value of the column weight is $CW_{\max}$.

(11) $$\begin{array}{c} Condition\;5:W_{i,j}\leq W_{\max},i=1\ldots m_{c},j=1\ldots n_{s}, \end{array}$$

where the weight of the element in the matrix is $W_{i,j}$ and the maximum value of non-zero element is $W_{\max}$.

The objective function of the differential evolution algorithm is formulated as

(12) $$\begin{array}{c} \min\psi \left(B_{J}\right),\\ s.t.\Omega \left(B_{J}\right)=1, \end{array}$$

where $\psi \left(B_{J}\right)$ denotes the joint source-channel decoding threshold and $\Omega \left(B_{J}\right)=1$ indicates that conditions 1–5 are satisfied.

Required algorithm parameters:

1. Number of population *P*.
2. Dimension *D* of the linking matrix $B_{L2}$ (i.e., the product of the number of rows and the number of columns) and the maximal value $W_{\max}$ of a single element in $B_{L2}$.
3. The maximum number of generations *G*.
4. Crossover probability value $p_{c}$.
5. Non-zero element probability Ep.
6. Non-zero element probability EDp.

Differential evolution algorithm is described as follows:

Step 1: Constructing a population *P* of dimension *D*, the number of elements of *D* is $m_{c}\cdot n_{s}$. The value of each element is less than or equal to $W_{max}$. Construct the population *P* repeatedly until each individual in the population satisfies conditions 1–5;

Step 2: The mutation produces the next generation under the following condition

(13) $$BM_{i}^{g+1}=B_{r1}^{g}+F\left(B_{r2}^{g}-B_{r3}^{g}\right).$$

In Equation (13), r1,r2,r3 are arbitrary values in [1,P], where [1,P] represents all individuals in the population of the differential evolution algorithm. $B_{r1}^{g}$, $B_{r2}^{g}$, and $B_{r3}^{g}$ denote randomly chosen individuals from the population, which are involved in the mutation process to generate the next generation individual $BM_{i}^{g+1}$. The values of $BM_{i}^{g+1}$ are rounded to integers, and *F* is the scaling factor;

Step 3: According to the probability $p_{c}$, crossover $BM_{i}^{g+1}$, and the previous $B_{i}^{g}$:

(14) $$B_{i}^{g+1}=\left\{\begin{array}{c} BM_{r1}^{g+1},\;\mathrm{if}\;\mathrm{rand}\;\left(0,1\right)\leq p_{c};\\ B_{r1}^{g},\;\mathrm{else}.\; \end{array}\right.$$

In Equation (14), $\;\mathrm{rand}\;\left(0,1\right)$ represents a randomly generated number between 0 and 1. This random number is used in the context of the crossover operation within the differential evolution algorithm. If the generated random number is less than the crossover probability $p_{c}$, the mutated individual is selected; otherwise, the parent individual is chosen for the crossover operation.

Step 4: Calculate the decoding threshold for every $B_{i}^{g+1}$, then select the decoding threshold that satisfies the requirements;

Step 5: Return to Step 2 until the calculation of the *G*th generation is completed, and the optimal decoding threshold is given.

The corresponding pseudocode of the algorithm is presented in Algorithm 1.
**Algorithm 1:** Design and optimization of $B_{L2}$ by differential evolution algorithm.**Require:**1:*P*: Population2:*B*: Linking matrix, number of rows $m_{c}$ and columns $n_{s}$3:$W_{max}$: Maximum value4:*D*: Dimension, $m_{c}$ multiplied by $n_{s}$5:*G*: Generation6:pc: Crossover probability7:Ep: Non-zero element probability**Ensure:**8:**Begin**9:**Initialization**10:**for**i=1:P **do**11:   **while** Condition 1–5 are not satisfied **do**12:     **for** j=1:D **do**13:        $e_{i}^{j}=rand\left(0,W_{max}\right)$;14:        ⊳ Random generate $B_{i}$. $e_{i}^{j}$ is the *j*-th element of $B_{i}$.15:     **end for**16:   **end while**17:**end for**18:**Calculation**19:**while** g≤G**do**20:   ⊳ *g* is initialized before its first use in the algorithm. This initialization ensures that *g* accurately records the generation count in the differential evolution process.21:   **for** i=1:P **do**22:     $B_{i}^{g+1}=$ Mutation $\left(B_{i}^{g}\right)$;23:     ⊳ The mutation function refers to the process described in Step 2 of the differential evolution algorithm.24:     Three individuals are randomly selected from the population, and the scaling factor is taken.25:     $B_{i}^{g+1}=\mathrm{Crossover}\left(B_{i}^{g+1},B_{i}^{g}\right)$;26:     ⊳ The crossover function refers to the process described in Step 3 of the differential evolution algorithm.27:     Crossover with probability pc.28:     **if** SatisfyCondition 1–5 **then**29:        **if** $\psi \left(B_{i}^{g+1}\right)\leq \psi \left(B_{i}^{g}\right)$ **then**30:          ⊳ψ(·) represents the calculation of the decoding threshold values.31:          $B_{i}^{g+1}\leftarrow B_{i}^{g+1}$;32:          ⊳$B_{i}^{g+1}$ has the minimum value of the JSCC decoding threshold.33:        **else**34:          $B_{i}^{g+1}\leftarrow B_{i}^{g}$;35:        **end if**36:     **end if**37:   **end for**38:   g=g+1;39:**end while**40:**End**

## 4. Results and Summary

**Figure 3:**
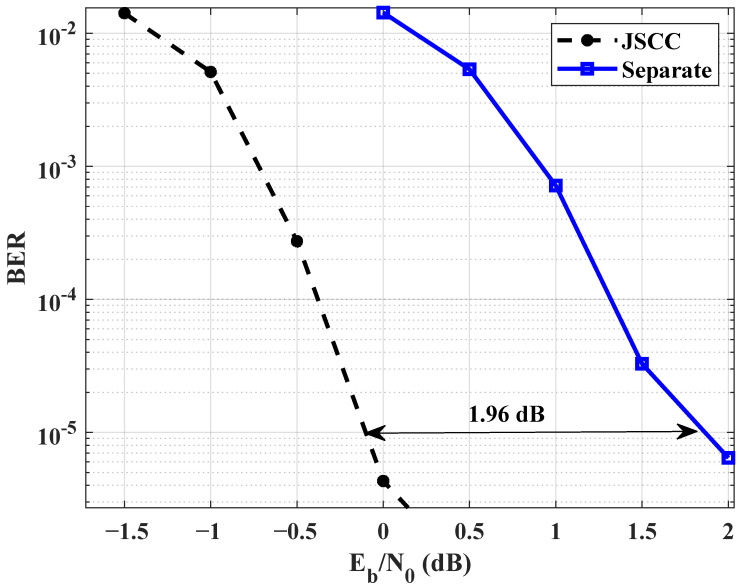
Comparison of BER values between the JSCC scheme and its separate scheme.

**Figure 4:**
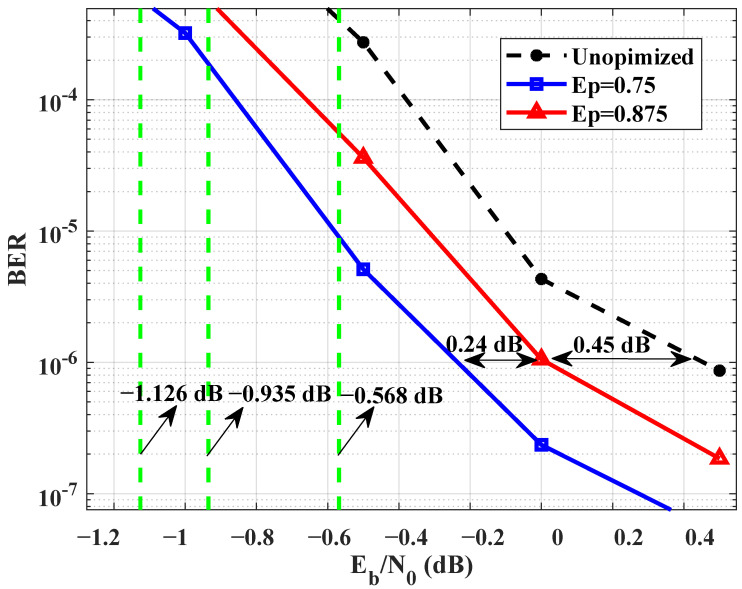
BER performance comparison of unoptimized $B_{J}^{\mathrm{UNO}}$, optimized $B_{J(0.75)}^{\mathrm{OPT}}$, and optimized $B_{J(0.875)}^{\mathrm{OPT}}$.

**Figure 5:**
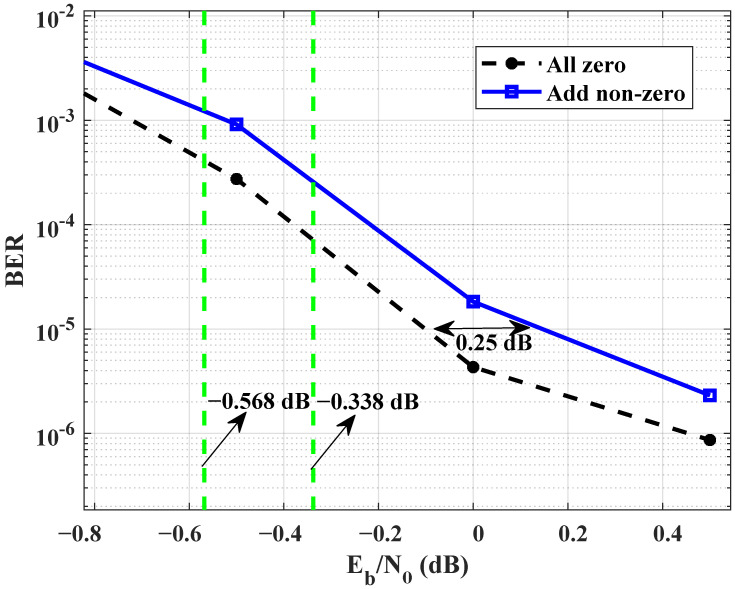
Comparison of BER values between the third row is all-zero elements and has non-zero elements.

**Figure 6:**
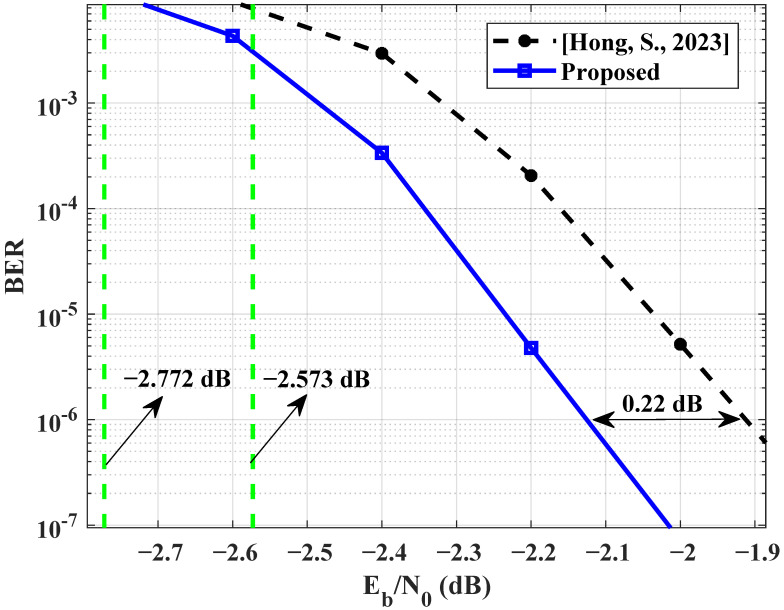
Comparison of BER values between [17] and the proposed optimization scheme.

The experiments in this paper were simulated under the AWGN channel. The source code and channel code were obtained via ’copy and permute’ from a progressive edge-growth (PEG) algorithm [18]. Simulation results were obtained by BPSK modulation and JBP iterative decoding. The maximum number of iterations was 100 and the maximum number of frames with errors was 100 for all the signal-to-noise ratios (SNRs). In Figure 3, Figure 4 and Figure 5, the source with p=0.02 is used, whereas in Figure 6, the source with p=0.04 is used. The value of $W_{\max}$ is set to 5 in order to reduce the space complexity of the differential evolution algorithm, which is O(P·D·G).

**Figure 7:**
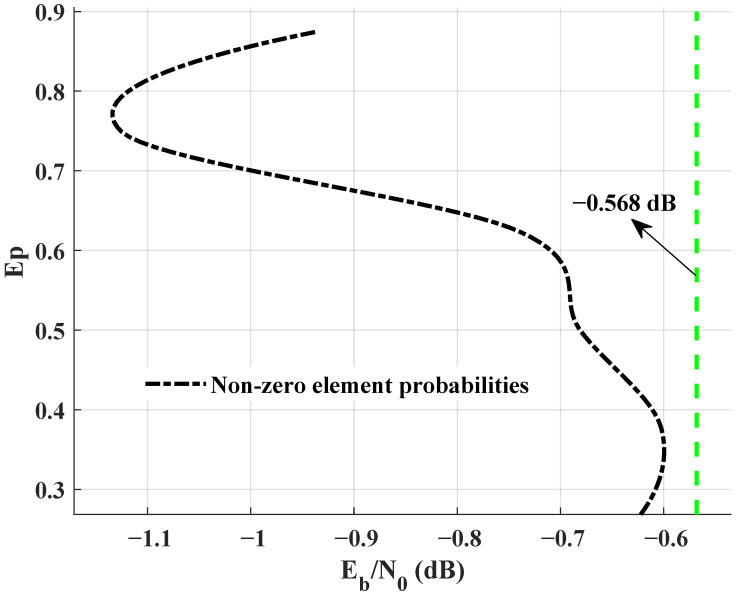
Decoding thresholds for different non-zero element probabilities in $B_{L2}$.

The study focuses on finding the ideal scheme with few calculations or approximating the ideal scheme at the minimum cost while constructing $B_{L2}$, because $B_{L2}$ has a unique effect on the performance of JSCC systems. $B_{L2}^{\mathrm{OPT}}$ is designed and optimized by differential evolution algorithm. Because the edge connections of the third row in $B_{L2}^{\mathrm{OPT}}$ may cause the performance degradation of the JSCC system, the elements of the third row are preset to zero. Non-zero elements with different probabilities are added to the first and second row of $B_{L2}$. The decoding threshold values corresponding to the probability of non-zero elements are depicted in Figure 7. When adding elements to a row with a probability of 0.875, it is necessary to ensure that the percentage of non-zero elements in at least one row of the matrix is at least 0.875, i.e. the percentage of elements in other rows is less than or equal to 0.875. The decoding threshold for this probability is −0.935 dB and the corresponding linking matrix is given by Equation (15). It is not the case that the more edges in linking matrix, the better the performance of the JSCC system.

(15) $$B_{J(0.875)}^{\mathrm{OPT}}=\left[\begin{array}{ccccccccccccc} 3 & 1 & 3 & 1 & 3 & 1 & 1 & 1 & 0 & 0 & 0 & 1 & 0\\ 1 & 2 & 1 & 3 & 1 & 3 & 1 & 2 & 0 & 0 & 0 & 0 & 1\\ 3 & 2 & 0 & 5 & 1 & 3 & 1 & 3 & 1 & 0 & 1 & 2 & 0\\ 1 & 1 & 1 & 3 & 0 & 2 & 1 & 2 & 0 & 1 & 0 & 3 & 1\\ 0 & 0 & 0 & 0 & 0 & 0 & 0 & 0 & 1 & 1 & 2 & 0 & 2 \end{array}\right]$$

(16) $$B_{J(0.75)}^{\mathrm{OPT}}=\left[\begin{array}{ccccccccccccc} 3 & 1 & 3 & 1 & 3 & 1 & 1 & 1 & 0 & 0 & 0 & 1 & 0\\ 1 & 2 & 1 & 3 & 1 & 3 & 1 & 2 & 0 & 0 & 0 & 0 & 1\\ 1 & 0 & 0 & 1 & 3 & 2 & 2 & 5 & 1 & 0 & 1 & 2 & 0\\ 1 & 3 & 0 & 0 & 2 & 0 & 1 & 0 & 0 & 1 & 0 & 3 & 1\\ 0 & 0 & 0 & 0 & 0 & 0 & 0 & 0 & 1 & 1 & 2 & 0 & 2 \end{array}\right]$$

A simulation performance comparison between JSCC and separate source-channel coding is presented in Figure 3. At a BER of $10^{-5}$, the JSCC system demonstrates a gain of 1.96 dB compared to its separate scheme. In order to improve the performance of the JSCC system, the maximum probability of non-zero elements in each row of $B_{L2}$ is 75 percent. The maximum number of the bits in a frame is 6400, i.e., the maximum length of the source sequence is 6400. The optimized matrix is in Equation (16). The decoding threshold value of $B_{J(0.75)}^{\mathrm{OPT}}$ is −1.126 dB, whereas the unoptimized linking matrix decoding threshold value is −0.568 dB. Thus, it yields a gain of 0.558 dB. Equation (16) gives that if there are too many non-zero elements in any row, it is necessary to reduce the number of non-zero elements in other rows to increase the overall system performance. Figure 4 shows BER performance comparison of unoptimized linking matrix, optimized $B_{J(0.75)}^{\mathrm{OPT}}$, and optimized $B_{J(0.875)}^{\mathrm{OPT}}$. At a BER level of $10^{-6}$, there has an approximately 0.69 dB gain between unoptimized linking matrix and optimized $B_{J(0.75)}^{\mathrm{OPT}}$. The performance of the optimized matrix $B_{J(0.875)}^{\mathrm{OPT}}$ gives to an about 0.45 dB gain.

To demonstrate the performance degradation of the waterfall region in JSCC system, the linking matrix with a decoding threshold of 0.338 dB in Table 1 was selected for the simulation experiment. Figure 5 presents a comparison of BER values between all-zero elements and non-zero elements in the third row. The simulation results indicate that adding unnecessary elements in the third row leads to performance degradation of the JSCC system. It is mainly because of the unnecessary non-zero elements, i.e., adding unnecessary edge connections, which disrupt the transmission of mutual information.

The proposed scheme for design and optimization of linking matrix is further verified. We have optimized the code pair $B_{J-\mathrm{new}\;1}^{0.04}$ in [17] when the probability of non-zero elements is 0.75. Figure 6 shows the BER performance of [17] and the optimized scheme. When the BER is $10^{-6}$, there is an approximate 0.22 dB gain between the proposed optimization scheme and that presented in [17]. This comparison experiment results verify the effectiveness of the proposed scheme for optimizing $B_{L2}$.

## 5. Conclusions

This paper discusses the impact of the linking matrix structure on the waterfall region of the JSCC system. Linking matrix with multi-edges can significantly improve the performance of a JSCC system, as compared to linking matrix with single-edge. However, effective principles are necessary for designing and optimizing linking matrix with multi-edges. An effective scheme for designing and optimizing linking matrix with multi-edges has been proposed based on the analysis of decoding thresholds. The proposed scheme achieves cost-effective design and optimization of the linking matrix and improves the performance of the JSCC system. Furthermore, JSCC exhibits brittleness, as it is optimized for particular combinations of source and channel coding and thus requires a redesign if either is changed.

## Figures and Tables

**Table 1:** Decoding thresholds (dB) of different elements in $B_{L2}$.

Degree	Threshold	Degree	Threshold	Degree	Threshold
$e_{L2}^{1,1}=5$	−0.598	$e_{L2}^{2,1}=5$	−0.566	$e_{L2}^{3,1}=5$	−0.479
$e_{L2}^{1,2}=5$	−0.505	$e_{L2}^{2,2}=5$	−0.443	$e_{L2}^{3,2}=5$	−0.401
$e_{L2}^{1,3}=5$	−0.613	$e_{L2}^{2,3}=5$	−0.576	$e_{L2}^{3,3}=5$	−0.483
$e_{L2}^{1,4}=5$	−0.568	$e_{L2}^{2,4}=5$	−0.513	$e_{L2}^{3,4}=5$	−0.466
$e_{L2}^{1,5}=5$	−0.593	$e_{L2}^{2,5}=5$	−0.587	$e_{L2}^{3,5}=5$	−0.482
$e_{L2}^{1,6}=5$	−0.556	$e_{L2}^{2,6}=5$	−0.518	$e_{L2}^{3,6}=5$	−0.465
$e_{L2}^{1,7}=5$	−0.453	$e_{L2}^{2,7}=5$	−0.431	$e_{L2}^{3,7}=5$	−0.338
$e_{L2}^{1,8}=5$	−0.536	$e_{L2}^{2,8}=5$	−0.515	$e_{L2}^{3,8}=5$	−0.403

## Data Availability

The data that support the findings of this study are available from the corresponding author upon reasonable request.
